# Dual use of antifungals in medicine and agriculture: How do we help prevent resistance developing in human pathogens?

**DOI:** 10.1016/j.drup.2022.100885

**Published:** 2022-10-20

**Authors:** Paul E. Verweij, Maiken C. Arendrup, Ana Alastruey-Izquierdo, Jeremy A.W. Gold, Shawn R. Lockhart, Tom Chiller, P.Lewis White

**Affiliations:** a Department of Medical Microbiology and Radboudumc-CWZ Center of Expertise for Mycology, Radboud University Medical Center, Nijmegen, the Netherlands; b Centre for Infectious Diseases Research, Diagnostics and Laboratory Surveillance, National Institute for Public Health and the Environment (RIVM), Bilthoven, the Netherlands; c Unit for Mycology, Statens Serum Insitut, Copenhagen, Denmark; d Department of Medical Microbiology, University Hospital Rigshospitalet, Copenhagen, Denmark; e Mycology Reference Laboratory, Instituto de Salud Carlos III, Madrid, Spain; f Mycotic Diseases Branch, Centers for Disease Control and Prevention, Atlanta, USA; g Public Health Wales Mycology Reference Laboratory, Cardiff, United Kingdom; h Department of Clinical Medicine, Copenhagen University, Copenhagen, Denmark

**Keywords:** *Aspergillus fumigatus*, DHODH inhibitor, One Health, triazole resistance, olorofim

## Abstract

Azole resistance in *Aspergillus fumigatus* is a One Health resistance threat, where azole fungicide exposure compromises the efficacy of medical azoles. The use of the recently authorized fungicide ipflufenoquin, which shares its mode-of-action with a new antifungal olorofim, underscores the need for risk assessment for dual use of antifungals.

Considering the interconnectedness of humans, animals, and the environment can foster our health and continued existence. Antimicrobial resistance (AMR) is a complex challenge that could benefit from a One Health perspective. In recent decades, AMR policies have focused on bacterial resistance and animal-environment-human interface, with limited attention to fungal resistance. However, the emerging threat of fungal resistance is increasingly recognized by public health institutes. The U.S. Centers for Disease Control 2019 Antibiotic Resistance Threats Report and the World Health Organization (WHO) Fungal Priority Pathogen List include *Candida auris* as an urgent public health threat (CDC, www.cdc.gov) and recently added azole-resistant *Aspergillus fumigatus* to the pathogen watchlist (WHO, www.who.int).

The dual use of azoles in the environment and in the clinic has been shown to be an important driver for azole-resistant *A. fumigatus* infections in humans (Snelders et al. 2012; Fisher et al., 2022). Recent surveillance studies show resistance rates varying between 1.4 % and 11.4 % in clinical *A. fumigatus* isolates, which predominantly (72 %, 95 % CI: 69–74) involve resistance mutations associated with environmental resistance selection (Table 1). Triazole fungicides and medical azoles share structural characteristics and therefore, mutations in the *cyp51A* target gene confer cross-resistance (Snelders et al., 2012). Although not a plant pathogen, *A. fumigatus* is environmentally ubiquitous and inevitably exposed to azole fungicides used for crop protection (Verweij et al., 2009). *A. fumigatus* thrives on decaying plant material and becomes resistant in the presence of azole fungicide residues (Schoustra et al., 2019). In addition to agricultural practices, substantial contamination of residential gardens with tebuconazole-resistant *A. fumigatus* was recently shown in a U.K.-wide study (Shelton et al., 2022). Migration of resistance through horticultural products and dispersal of *Aspergillus* conidia with air currents has facilitated global spread and make control difficult (Dunne et al., 2017; Fisher et al., 2022). Adaptation to azole stress may also drive other adaptations potentially leading to increased virulence in host infection (Fisher et al., 2022). Current clinical implications of azole resistance are significant with 25 % day-90 excess mortality in patients with voriconazole-resistant invasive aspergillosis who were treated with voriconazole (Lestrade et al., 2019). Azoles are the first-line treatment and currently the only oral option for *A. fumigatus* infections, and few alternative treatment options exist for azole-resistant aspergillosis (Ostrosky-Zeichner et al., 2010; Verweij et al., 2015).

Developing antifungal drugs is challenging because of the homology of drug targets between fungal and human eukaryotic cells (Ostrosky-Zeichner et al., 2010). However, researchers have recently discovered the orotomide class, which targets the biosynthesis of pyrimidines by inhibiting the key enzyme dihydroorotate dehydrogenase (DHODH) (Oliver et al., 2016). Olorofim, an orally available drug currently undergoing clinical evaluation, is the first member of its class to show promising in vitro and in vivo activity against azole-resistant *A. fumigatus* and other difficult-to-treat mold infections (Jørgensen et al., 2018; Hoenigl et al., 2021). The U.S. Food and Drug Administration granted breakthrough drug therapy designation and orphan drug designation to olorofim for the treatment of invasive aspergillosis (FDA, www.fda.gov), reflecting the potential improvement the drug may offer over existing therapies.

We can learn from the One Health azole-resistant *A. fumigatus* challenge to preserve the clinical efficacy of new modes of action (MoA). Evaluating the use of similar compounds in medical and non-medical applications and incorporating an assessment of risk to human and animal health during the authorization process could prevent an environmental route of resistance selection for novel classes. Of concern is the use of the recently authorized fungicide ipflufenoquin (EPA, www.epa.gov). The Fungicide Resistance Action Committee (FRAC) has classified ipflufenoquin as a DHODH inhibitor, meaning ipflufenoquin shares its MoA with olorofim (FRAC, www.frac.info). Furthermore, the risk of resistance selection by ipflufenoquin is considered moderate to high, suggesting a potential human health concern.

Preliminary in vitro susceptibility testing of 21 *Aspergillus* isolates indicated that olorofim and ipflufenoquin display in vitro activity against all but four and five *Aspergillus* spp. isolates, respectively (Table 2). With all four isolates in Section *Aspergillus* demonstrating high MIC values to olorofim and 3/4 of these isolates also potentially resistant to ipflufenoquin which also appeared to be ineffective against *A. niger* at both 50 % and 100 % growth inhibition endpoints (Table 2)(Materials and methods are shown in supplementary material). In general, a good correlation was observed between the in vitro activity against *Aspergillus* spp., with *A. sydowii, A. flavus* and *A. terreus* species complex (sc) being most susceptible and *A. nidulans* sc, *A. niger* sc, *A. calidoustus,* Section *Aspergillus* being the least susceptible species. This was not the case comparing MICs against 16 other mold spp. with the exception of both agents being inactive against the *Rhizopus microsporus,* in agreement with the DHODH drug target being absent in Mucorales (Table S1).

On the basis of drug concentration (mg/L), olorofim was more potent against *Aspergillus* (MIC_50_ 0.016/0.06 mg/L with 50 % and complete inhibition endpoints, respectively) than ipflufenoquin (MIC_50_ 2/4 mg/L). With the difference slightly larger for *A. fumigatus* sensu stricto (MIC_50_ 0.016/0.06 mg/L for olorofim versus MIC_50_ 2/16–32 mg/L for ipflufenoquin). Our data suggest an overall similarity between the medical DHODH inhibitor olorofim and the agricultural pesticide ipflufenoquin against *Aspergillus* spp., supporting the concern that ipflufenoquin use may cause cross-resistance to olorofim in *Aspergillus.* However, the ability of ipflufenoquin to select for olorofim resistance and associated mutations in the target *PyrE*-gene would need to be demonstrated. If confirmed, environmental monitoring programs are needed to determine if resistance selection in *A. fumigatus* occurs in the field.

In addition to orotomides, other new MoA’s are in clinical development, including fosmanogepix, an inhibitor of fungal protein Gwt1. Pyridine fungicides that target this same enzyme, such as aminopyrifen, are in development with a potential risk for cross resistance emerging in human fungal pathogens (Hatamoto et al., 2019).

Increased emphasis and inclusion of antifungal resistance by research and surveillance programs could help inform policymakers about the risks and benefits of antifungal applications. For example, global action plans involving the quadripartite WHO, Food and Agriculture Organization of the United Nations, World Organization for Animal Health, and the UN Environment Progamme, could address risk assessment strategies for new MoA in relation to the development of cross-resistance in medically relevant (non-target) fungi. International surveillance programs could detect and monitor trends in AMR, especially those taking a combined One Health approach, but need to incorporate antifungal resistance. The incorporation of fungi in the Strategic Research and Innovation Agenda of the Joint Programming Initiative on AMR (JPIAMR) represents a first step to address urgent questions related to fungal AMR (Fisher et al., 2022). Leading international mycology societies, including the European Confederation for Medical Mycology (ECMM), the International Society for Human and Animal Mycology (ISHAM), the Mycoses Study Group Education and Research Consortium (MSGERC), and the ESCMID Fungal Infection Study Group (EFISG), have endorsed the need to prioritize One Health antifungal resistance research and policies that protect antifungal drugs developed for treatment of fungal diseases and to incorporate these into the early steps of fungicide development. Furthermore, fungicide producers are also stakeholders that need to take responsibility for safeguarding crop protection without unintended effects for human health. In order to complete our understanding of the One Health perspective, it is imperative that they are involved in future discussions on managing this critical issue. Together, these actions may help to curb fungal AMR on a global scale.

## Supplementary Material

supplemental materials

## Figures and Tables

**Table 1:** Azole resistance rates in clinical *A. fumigatus* isolates in recent surveillance studies.

Continent	Country; Years	Prevalence^a^	Characteristics^b^	Reference
Europe	Denmark; 2018–2020	6.1 % (66/1083)	National surveillance TR_34_ (39/66; 59 %)	Risum et al., 2022
	Belgium; 2016–2020	7.1 % (85/1192)	Single center TR_34_ (74/78; 95 %)	Resendiz-Sharpe et al., 2021
	Netherlands; 2018–2021	9.2 % (567/6134)	Multicenter (10 centers) TR_34_ (392/660; 59 %); TR_46_ (132/660; 20 %)	https://swab.nl/nl/nethmap
	Turkey; 2018–2019	3.3 % (13/392)	Multicenter (21 centers) TR_34_ (9/19; 47 %)	Ener et al., 2022
	France; 2017	2.1 % (4/195)	Single center No TR resistance mechanisms	Simon et al., 2021
	Switzerland; 2018–2019	1.4 % (3/365)	Multicenter (7 centers) TR_34_ (3/3; 100 %)	Ragozzino et al., 2021
	Italy; 2016–2018	6.6 % (19/286)	Multicenter, isolate denominator TR_34_ (12/19; 63 %)	Prigitano et al., 2021
	Spain; 2019	4.7 % (34/725)	Multicenter TR_34_ (19/34; 56 %); TR_46_ (1/34; 3 %)	Escribano et al., 2021
Americas	USA; 2015–2020	3.5 % (73/2072)	Multicenter, isolate denominator TR_34_ (7/73; 10 %); TR_46_ (7/73; 10 %)	Badali et al., 2022
	Martinique; 2014–2018	11.4 % (4/35)	Single center, isolate denominator TR_34_ (1/4; 25 %)	Monpierre et al., 2021
Asia	China; 2019–2020	4.1 % (3/73)	Single center, patients with IA. TR_46_ (2/3; 67 %)	Wang et al., 2022
	China; 2009–2019	4.3 % (19/445)	Single center TR_34_ (4/19; 21 %); TR_46_ (3/19; 16 %)	Yang et al., 2021
	China; 2016–2018	7.0 % (4/57)	Two hospitals TR_46_ (1/4; 25 %)	Xu et al., 2020
	Taiwan; 2015–2020	1.8 % (2/113)	Single center TR_34_/L98H (2/2; 100 %)	Hsu et al., 2022

a Number of patients with a positive *A. fumigatus* is used as denominator, unless stated otherwise.

b TR_34_ and TR_46_ refer to the genetic background of the resistance mutations. IA, invasive aspergillosis.

**Table 2:** Comparison of the in vitro activity of two DHODH inhibitors, olorofim and ipflufenoquin against *Aspergillus* species determined according to the EUCAST E.Def 9.4 with a complete inhibition endpoint and by applying a partial (50%) growth inhibition endpoint.

Inhibition endpoint criteria and species (*n*)	Olorofim MIC (mg/L)												Ipflufenoquin MIC (mg/L)									
0.002	0.004	0.008	0.016	0.03	0.06	0.125	0.25	0.5	1	2	> 4	0.06	0.125	0.25	0.5	1	2	4	8	16	32	> 32
Visual complete inhibition																						
*A. sydowii* (2)		1	1									2										
*A. flavus* SC (2)		1	1										1	1								
*A. terreus* SC (2)			2												1	1						
*A. fumigatus* SC* (5)			1	1	3												1**	1**	1	2		
*A. nidulans* SC (2)					2											2						
*A. niger* SC (2)				1	1																2	
*A. calidoustus* (2)							2								2							
Section *Aspergillus*# (4)												4								1		3
Spec. 50% inhibition																						
*A. sydowii* (2)		1	1									1	1									
*A. flavus* SC (2)	1		1										2									
*A. terreus* SC (2)	1	1										1				1						
*A. fumigatus* SC * (5)			1	4												4		1				
*A. nidulans* SC (2)				2											1	1						
*A. niger* SC (2)				1	1																2	
*A. calidoustus* (2)							1	1							1	1						
Section *Aspergillus*# (4)												4						1##				3

# One *A. montevidensis*, one *A. chevalieri*, two *A. intermedius*.

## one *A. chevalieri*.

* Two *A. fumigatus* Cyp51A wild-type, one *A. fumigatus* Cyp51A TR_34_/L98H, and two *A. hiratsukae*.

** Two *A. hiratsukae*.
