# Supplementary material for: Dual use of antifungals in medicine and agriculture: How do we help prevent resistance developing in human pathogens?
Source: Drug Resist Updat. Author manuscript; Available in PMC 2023 Dec 3. (PMC10693676; doi:10.1016/j.drup.2022.100885)
Supplement: supplemental materials [file NIHMS1943354-supplement-supplemental_materials.docx]

**Supplement**

**Materials and Methods**

A total of 37 mould isolates from 20 species were included. Twenty-one were *Aspergillus* spp. isolates (15 species) whereas 16 were other moulds from nine different species. The isolates were cultured from clinical samples during 2018-2020. Susceptibility testing was performed according to the EUCAST E.Def 9.3 reference method and MICs read visually according to the standard full inhibition endpoint and spectrophotometric using 90% and 50% growth inhibition as compared to the growth control yielding 333 MICs (Arendrup et al., 2016).

Stock solutions of antifungal agents were prepared in DMSO and twofold dilutions prepared according to the ISO method (Arendrup et al., 2021). The following final concentration ranges (manufacturer) were used: olorofim 4 - 0.001 mg/L, ipflufenoquin (Kinoprol, Nippon Soda Co., Ltd.) and tetflupyrolimet (FMC Corp Delaware, USA) 32 - 0.03 mg/L.

**References**

Arendrup MC, Meletiadis J, Mouton JW, Guinea J, Cuenca-Estrella M, Lagrou K, Howard SJ. EUCAST technical note on isavuconazole breakpoints for Aspergillus, itraconazole breakpoints for Candida and updates for the antifungal susceptibility testing method documents Clin Microbiol Infect 2016 Jun;22(6):571.e1-4. doi: 10.1016/j.cmi.2016.01.017. Epub 2016 Feb 3.

Arendrup MC, Jørgensen KM, Hanemaaijer N, Verweij PE. ISO standard 20776-1 or serial 2-fold dilution for antifungal susceptibility plate preparation: that is the question! J Antimicrob Chemother 2021; 76(7): 1793–1799, https://doi.org/10.1093/jac/dkab088

**Table S1.** Comparison of the *in vitro* activity of two DHODH inhibitors, olorofim (human fungicide) and ipflufenoquin (agricultural pesticide) against mould species other than *Aspergillus* determined according to the EUCAST E.Def 9.4.

| **Inhibition endpoint**  **criteria and species (*n*)** | **Olorofim MIC (mg/L)** | | | | | | | | | | | |  | **Ipflufenoquin MIC (mg/L)** | | | | | | | | | | | |
| --- | --- | --- | --- | --- | --- | --- | --- | --- | --- | --- | --- | --- | --- | --- | --- | --- | --- | --- | --- | --- | --- | --- | --- | --- | --- |
|  | **0.002** | **0.004** | **0.008** | **0.016** | **0.03** | **0.06** | **0.125** | **0.25** | **0.5** | **1** | **2** | **>4** |  | **≤0.03** | **0.06** | **0.125** | **0.25** | **0.5** | **1** | **2** | **4** | **8** | **16** | **32** | **>32** |
| **Spec. 50% inhibition** |  |  |  |  |  |  |  |  |  |  |  |  |  |  |  |  |  |  |  |  |  |  |  |  |  |
| *Rasamsonia argillacea* (1) |  | 1 |  |  |  |  |  |  |  |  |  |  |  |  |  | 1 |  |  |  |  |  |  |  |  |  |
| *Rasamsonia aegroticola* (1) | 1 |  |  |  |  |  |  |  |  |  |  |  |  |  |  | 1 |  |  |  |  |  |  |  |  |  |
| *Fusarium proliferatum* (2) |  |  |  | 2 |  |  |  |  |  |  |  |  |  |  |  | 2 |  |  |  |  |  |  |  |  |  |
| *Scedosporium*## (4) |  | 1 |  | 2 |  | 1 |  |  |  |  |  |  |  | 4 |  |  |  |  |  |  |  |  |  |  |  |
| *Paecilomyces variotii* (2) |  |  |  |  | 2 |  |  |  |  |  |  |  |  |  |  |  |  |  | 1 |  | 1 |  |  |  |  |
| *Purpureocillium lilacinum* (2) |  |  |  |  |  |  |  |  |  | 2 |  |  |  | 2 |  |  |  |  |  |  |  |  |  |  |  |
| *Fusarium solani* (2) |  |  |  |  |  |  |  |  |  | 1 | 1 |  |  |  | 1 | 1 |  |  |  |  |  |  |  |  |  |
| *Rhizopus microsporus* (2) |  |  |  |  |  |  |  |  |  |  |  | 2 |  |  |  |  |  |  |  |  |  |  |  |  | 2 |
| **Visual complete inhibition** |  |  |  |  |  |  |  |  |  |  |  |  |  |  |  |  |  |  |  |  |  |  |  |  |  |
| *Rasamsonia argillacea* (1) |  |  | 1 |  |  |  |  |  |  |  |  |  |  |  |  |  |  | 1 |  |  |  |  |  |  |  |
| *Rasamsonia aegroticola* (1) |  |  |  | 1 |  |  |  |  |  |  |  |  |  |  |  |  | 1 |  |  |  |  |  |  |  |  |
| *Fusarium proliferatum* (2) |  |  |  |  | 1 | 1 |  |  |  |  |  |  |  |  |  |  |  | 1 | 1 |  |  |  |  |  |  |
| *Scedosporium*## (4) |  |  |  |  | 2 | 1 | 1 |  |  |  |  |  |  | 4 |  |  |  |  |  |  |  |  |  |  |  |
| *Paecilomyces variotii* (2) |  |  |  |  |  |  | 1 | 1 |  |  |  |  |  |  |  |  |  |  |  |  |  |  |  |  | 2 |
| *Purpureocillium lilacinum* (2) |  |  |  |  |  |  |  |  |  |  |  | 2 |  | 1 | 1 |  |  |  |  |  |  |  |  |  |  |
| *Fusarium solani* (2) |  |  |  |  |  |  |  |  |  |  |  | 2 |  |  |  |  |  |  | 2 |  |  |  |  |  |  |
| *Rhizopus microsporus* (2) |  |  |  |  |  |  |  |  |  |  |  | 2 |  |  |  |  |  |  |  |  |  |  |  |  | 2 |

## Two *S. boydii* and two *S. apiospermum*
